# The Association Between the 5Cs and Anxiety—Insights From Three Countries: Portugal, Slovenia, and Spain

**DOI:** 10.3389/fpsyg.2021.668049

**Published:** 2021-06-02

**Authors:** Ana Kozina, Diego Gomez-Baya, Margarida Gaspar de Matos, Gina Tome, Nora Wiium

**Affiliations:** ^1^Educational Research Institute, Ljubljana, Slovenia; ^2^Department of Social, Developmental and Educational Psychology, Universidad de Huelva, Huelva, Spain; ^3^FMH/ISAMB Universidade de Lisboa, Lisbon, Portugal; ^4^University of Bergen, Bergen, Norway

**Keywords:** anxiety, adolescence, 5Cs, Portugal, Slovenia, Spain

## Abstract

Several of the most frequent psychological difficulties in childhood and adolescence are related to anxiety and lead to numerous short- and long-term negative outcomes in emotional, social, and academic domains. Empirical evidence consistently shows that the 5Cs (competence, caring, confidence, connection, and character) of Positive Youth Development (PYD) are positively related to adolescents’ contribution to self, family, and society as well as negatively related to risky behaviors and emotional difficulties, such as anxiety. Thus, the PYD can be one of the models that informs prevention programs. To provide contextualized, data-driven support for prevention efforts, we have analyzed the predictive value of the 5Cs for anxiety and anxiety dimensions using three different convenience youth samples from Portugal (*N* = 384, 46.6% female), Slovenia (*N* = 449, 69% females), and Spain (*N* = 768; 60.5% females). To assess the 5Cs, we used the same short form of the PYD scale in all samples (Geldhof et al., 2013) and different anxiety measures across samples: the Multidimensional Anxiety Scale for Children (MASC) in Portugal, the Lestvica anksioznosti za otroke in mladostnike anxiety scale (LAOM) in Slovenia and the Generalized Anxiety Disorder-7 (GAD-7) in Spain. The findings show significant associations of PYD and anxiety across all three contexts with all three different anxiety measures used. The associations vary across countries emphasizing the need to further research the role of contexts in anxiety prevention. Despite variations the results do indicate that connection is negatively associated with anxiety in all three contexts using the three anxiety measures, while confidence is a negative predictor and caring is a positive predictor of anxiety in Slovenia and Spain. Implications for practice within an educational framework for adolescents and youth are discussed, together with public policy recommendations.

## Introduction

Anxiety-related difficulties are one of the most frequent psychological challenges in childhood and adolescence (Neil and Christensen, 2009). These difficulties trigger numerous short- and long-term negative outcomes related to social, emotional, and cognitive functioning that result in leaving school early, need for mental health care, work deficiency, lower life satisfaction later in life (Weems and Stickle, 2005), and are on the rise (Twenge, 2000; Kozina, 2014). Among other negative consequences (in emotional, social, and academic domains), anxiety disorders (i.e., anxiety in its severe forms) are a risk factor in the development of suicidal ideation, mood, and substance use disorders (Hofstra et al., 2002; Sareen et al., 2005). Even though anxiety is common in childhood and adolescence, it becomes problematic when it is persistent, frequent, and severe enough to restrain the child or adolescent in his or her daily functioning (Weems and Stickle, 2005). It is estimated that globally, severe anxiety affects as many as 20% of youth (Costello et al., 2011). Despite the importance of intervention, only a fraction of youth with anxiety disorders (about 30%) receives treatment (Merikangas et al., 2011). To enhance the effectiveness of prevention and intervention programs, theoretically sound models with clear links between protective factors and outcomes are needed (Silverman and Treffers, 2001), especially in childhood and adolescence, when the onset of anxiety disorders typically occurs (Kessler et al., 2005). One of the models that can serve as a theoretical framework in designing prevention and intervention programs targeting anxiety is the Positive Youth Development (PYD) perspective (Lerner, 2007).

The PYD is embedded in the Relational Development Systems Model (Overton, 2015), with the focus on the importance of the interaction that takes place between the individual and his or her context (e.g., school, family, community, and society; Lerner, 2007). The Relational Developmental System Model (Overton, 2015) argues that young people should be studied as the product of a mutually reinforcing interaction between individual characteristics (internal assets) and youth contextual resources (external assets)—the so-called adaptive developmental regulations (Lerner et al., 2006). As a result of the adaptive development regulations, PYD outcomes (typically defined as the 5Cs: competence, confidence, character, caring, and connection) are facilitated. Confidence is defined as an inner feeling of positive self-worth and self-efficacy. Competence is a positive view of one’s own actions in specific areas (e.g., social and academic skills). Connection stands for all positive mutual ties that an adolescent has with significant others and institutions. Character is defined as the possession of standards for correct behavior in relation to social and cultural norms. A feeling of sympathy and empathy for others indicates caring. There is evidence that components of the 5Cs are positively related to an adolescent’s contribution to his- or herself, his or her family, and society, as well as negatively related to risky behaviors and emotional difficulties, such as anxiety.

Following the theoretical model, a negative association between the 5Cs and anxiety is expected. Nevertheless, research shows that the association between the 5Cs and positive outcomes depends on whether the 5Cs are treated as a global factor or as distinct components of PYD. There is a theoretical assumption (with empirical support) that the 5Cs reflect a global PYD factor (Lerner et al., 2005; Phelps et al., 2009; Bowers et al., 2010), while the 5Cs are also distinct components (Geldhof et al., 2014; Erentaitë and Raižienë, 2015). When a global PYD factor is examined, there is a negative association between PYD and anxiety (Geldhof et al., 2014; Erentaitë and Raižienë, 2015; Holsen et al., 2017). By contrast, when the 5Cs are studied as distinct components, not all are negatively associated with anxiety. For example, caring has been found to be positively associated with anxiety in several studies (Geldhof et al., 2014; Truskauskaitë-Kunevičienë et al., 2014; Holsen et al., 2017) and also in a Slovenian youth sample (Kozina et al., 2020). Possible mechanisms underlying this positive association between caring and anxiety have been provided by Geldhof et al. (2019). The authors explain that the relationship between an individual and his or her context is not always positive (e.g., adaptive developmental regulations); it can also be neutral or negative. Negative developmental regulations can take place where the process can either harm the individual (self-sacrificing developmental regulations, martyrdom developmental regulations), or the context (parasitic developmental regulations), or both (maladaptive developmental regulations). There is evidence that PYD indicators such as the 5Cs promote positive outcomes only when they exist as part of mutually beneficial relationships between the individual and his or her unique contexts (adaptive developmental regulations), and that there is also a conditional relationship between the 5Cs and healthy development (Holsen et al., 2017; Geldhof et al., 2019). This makes research on the role context play in these relationships even more important. To add to the complexity, anxiety, like the PYD, is multidimensional. It is a combination of cognitive (e.g., worries), physiological (e.g., nausea), emotional (e.g., fear), and behavioral (e.g., avoidance) responses (Silverman and Treffers, 2001). Research shows that different dimensions of anxiety show different stability (e.g., the physical dimension is more stable; Olatunji and Cole, 2009) and different components are reflected in different anxiety disorders (e.g., the physical dimension is associated with panic disorder; McLeod et al., 2011). This is why a multidimensional analysis of anxiety provides an added value when looking at its associations with the PYD across different age samples.

### The Contexts

Anxiety, like the 5Cs, depends on the wider context. More specifically, the characteristics of society that influence anxiety are (a) the level of overall threat, (b) economic conditions, and (c) social connectedness (Twenge, 2000). Thus, it is important to study anxiety, as multidimensional construct, together with its associated factors across different contexts (e.g., countries, educational levels, developmental stages). The three contexts in question—Portugal, Slovenia, and Spain—are all dealing with high anxiety (and depression) rates while at the same time using different prevention and intervention methods.

In Portugal mental health statistics have shown negative trends in the last decade, with 20% of adolescents and young adults presenting psychopathological disorders (Marques and Brissos, 2014) and suicide as one of the leading causes of death among Portuguese youth (Marques and Brissos, 2014). There are several interventions based on PYD that seek to promote the 5Cs in school contexts. PYD initiatives in school setting are aiming (a) to promote interpersonal communication and problem-solving competences (de Matos et al., 2012); (b) to train teachers to be able to foster children and adolescents’ competences, well-being, and mental health (Tomé et al., 2018, 2019a); (c) to adopt a “whole school approach (Tomé et al., 2021); and (d) to focus on young people’s engagement and social participation (Branquinho et al., 2019, 2021).

Similarly, mental health statistics for Slovenia reveal disturbing trends of high suicide rates in the general population (e.g., 20 per 100 000 suicides in the last decade) (Roškar et al., 2020) and in Slovenian youth (above European average, Jeriček Klanšček et al., 2018) with suicide ranking as one of the three main causes of death in Slovenia (Jeriček Klanšček et al., 2018). Anxiety is a risk factor for suicide (Abreu et al., 2018) and in Slovenia there is documented increase in treated anxiety disorders from 2008 to 2014. This trend is even more evident in older adolescents (15–19 years) compared to younger adolescents (6–14 years) and in girls compared to boys (Jeriček Klanšček et al., 2018). High suicide rates among youth together with growing unemployment rates and a heightened risk of poverty among young people (Senekovič, 2016), makes the need for anxiety prevention programs in youth critical. Unfortunately, in Slovenia PYD-related intervention is not as systematic as it is in Portugal. The systemic support for anxiety prevention is lacking and PYD remains an emerging field of research (Gonzalez et al., 2017; Kozina et al., 2019). There are, however, several small-scale initiatives supporting psychological well-being and promoting social and emotional competencies (e.g., HAND in HAND, Kozina, 2020; FRIENDS, Kozina, 2018; and To Sem Jaz; Tacol et al., 2019), but they are (a) not PYD based, (b) lack systemic support, and (c) are usually implemented in primary and lower secondary schools and not in upper secondary schools or faculties.

In Spain, data from the National Health Survey (Spanish Health Ministry, 2017) showed a prevalence of 7.36% of chronic anxiety in the overall population, with 2.36% observed in people aged 15–24 years and 5.32% in people aged 25–34 years. Furthermore, based on the Barometer on Youth Life and Health (Queen Sofia Center, 2017), 11% of the population aged 15–29 years old experienced anxiety, panic, or phobia. Concerning PYD experiences in Spain, very few studies and interventions have been conducted to date, although they have offered promising results (Pertegal et al., 2010; Gomez-Baya et al., 2019). In childhood and adolescence, the Happy Classrooms Programme presents a positive education experience with sessions to build character strengths and practice mindfulness in order to promote well-being and positive school climate (Arguis et al., 2010). This intervention contains more than 300 activities adapted to different levels of education (i.e., childhood education, primary education and secondary education). Lombas et al. (2019) tested a brief version of this program in a sample of adolescents and proved its effectiveness to promote satisfaction with life, self-esteem, emotional intelligence, relatedness, affiliation and teachers’ support, self-regulation and mindfulness, and to reduce perceived stress, depression, amotivation, and aggression. Furthermore, the Healthy Universities Network has been recently created, composed of 60 public and private universities, in order to enhance healthy lifestyles and psychological well-being among undergraduates (Red Española de Universidades Saludables, 2018). In this network, some working groups have been developed to: (a) define the assessment criteria for health diagnostic in the university community, (b) design online training about healthy lifestyles for undergraduates, (c) examine addiction to new technologies, (d) describe the indicators for a healthy university campus, and e) design a promotional video and leaflet to advertise the network. Thus, age-specific interventions within school or university contexts may be recommended, adapted to different developmental characteristics of the populations and concrete mental health needs. More research and practice are needed from PYD theory in Spain, and anxiety appears to be an important target for interventions with adolescents and youth.

In all three contexts, Portugal, Slovenia, and Spain, anxiety is highly prevalent and in need for intervention. The systemic support for anxiety prevention and intervention differs substantially with Portugal being in a better position. In Portugal, PYD intervention has already been implemented and evaluated, while the PYD interventions in Slovenia and Spain are still based on small scale initiatives and research project. There are also some differences in target population of adolescents and youth. In Slovenia and Portugal, the focus is on primary and lower-secondary educational level, while Spain shows promising initiatives also at the faculty level.

### Current Study

Our research questions are based on a previous research that identified a positive association between one component of PYD, that is caring, and anxiety in Slovenia (Kozina et al., 2020). We are interested in the differential role of a global PYD factor and anxiety as well as the different components of the 5Cs and anxiety in Slovenia and two additional contexts, Portugal and Spain. We used three different anxiety measures (clinical and non-clinical, multi-dimensional, one-dimensional) in three different contexts (Portugal, Slovenia, and Spain) as outcomes and the 5Cs and the global PYD factor as predictors. With the use of different anxiety measures in different (not-directly comparable) samples the findings have the potential to inform developmental effects on the relationship between PYD and anxiety.

Our research explores:

(a) The association between a global PYD factor and anxiety (and its dimensions) vs. the association between components of the 5Cs and anxiety (and its dimensions);
(b) the predictive power of the 5Cs for different dimensions of anxiety (non-clinical: *emotions*, *worries*, and *decision*; clinical: *physical symptoms*, *harm avoidance*, *social anxiety*, and *separation anxiety*); and
(c) whether these associations are different or similar in different countries.

Based on the PYD theoretical model (Lerner, 2007), we expect negative relationship between PYD global score and anxiety in all three contexts. In addition, based on our previous research using Slovenian data (Kozina et al., 2020), we expect similar pattern of associations in Portuguese and Spanish samples. More specifically we expect positive association between caring and anxiety and a negative association between the other four Cs (competence, confidence, character, and connection) and anxiety. We expect the patterns of associations to be similar across contexts, even though not directly comparable due to the different measures used.

## Materials and Methods

### Participants

In Portugal, a convenience sample of adolescents (*N* = 384, 46.6% female) was involved in the study; they were between 10 and 20 years old (*M*_*age*_ = 15.3, *SD* = 2.3), 13% (*n* = 50) aged between 10 and 12 years, 36.5% (*n* = 140) between 13 and 15 years and 50.5% (*n* = 194) over 16 years. Participants were 5th to 12th grade students attending public schools (lower and upper secondary level), and 57% (*n* = 220) attending a secondary school (upper-secondary level). In Slovenia, a convenience sample of adolescents (*N* = 449, 69% females) aged between 15 and 23 years (*M*_*age*_ = 16.96, *SD* = 1.59, 50.4% aged between 15 and 16 years, 74.3 living with both parents; 59.1% living in small town; 33% had mothers with university degree, 23.8 had fathers with university degree), who were enrolled in upper secondary schools, were recruited. In Spain, a convenience sample (*N* = 768 adolescents; 60.5% females) aged between 17 and 29 years (*M*_*age*_ = 19.50, *SD* = 2.27) was enrolled in 10 educational institutions, three universities (tertiary level) and seven high schools (upper-secondary level), in Andalusia. About 60% of the institutions were private and 40% were public, with 60% located in urban areas and 40% in rural areas. Participating classes within each institution were then selected at random. Regarding age of participants, 42.1% were aged 17–18 years old, 44.1% aged 19–21, and 13.8% aged 22–29. Furthermore, 70.8% lived with both parents, 74.6% lived in a city, and over 35% had mothers/fathers with university degree. Most of the sample were born in Spain (95.8%).

### Measures

The short form of the PYD questionnaire (Geldhof et al., 2013), comprising 34 items, was used to assess the 5Cs, with responses given on a 5-point Likert scale from 1 (strongly disagree) to 5 (strongly agree). Sample items are: Competence (e.g., “I do very well in my classwork at school”), Confidence (e.g., “All in all, I am glad I am me”), Caring (e.g., “When I see another person who is hurt or upset, I feel sorry for them”), Character (e.g., “I hardly ever do things I know I shouldn’t do”), and Connection (e.g., “My friends care about me”). In all three language versions the original items were translated using committee approach. In Portugal, the dimensions Caring (α = 0.87), Confidence (α = 0.87), Connection (α = 0.81), Competence (α = 0.80), and Character (α = 0.73) showed acceptable internal consistency. In a Portuguese version (Tomé et al., 2019b), a five-factor structure presented an adequate fit based on confirmatory factor analysis (CFA), χ^2^ = (509) = 960.19, *p* < 0.001, comparative fit index (CFI) = 0.90, root mean square error of approximation (RMSEA) = 0.04. In Slovenia, the PYD questionnaire has proven to be psychometrically adequate with reliability coefficients as follows: α = 0.78 (Competence), α = 0.82 (Confidence), α = 0.74 (Character), α = 0.91 (Caring), and α = 0.81 (Connection). CFA using a Slovenian version confirmed a good fit of the five-factor structure, χ^2^ (517) = 8745.158, *p* < 0.001, RMSEA = 0.063, 90% CI [0.062, 0.065], CFI = 0.947, Tucker–Lewis index (TLI) = 0.942 (Gonzalez et al., 2017). In Spain, as shown in Gomez-Baya et al. (2019), the overall PYD scale presented good internal consistency (α = 0.86). The dimensions of Confidence (α = 0.74), Connection (α = 0.73) and Caring (α = 0.86) showed acceptable internal consistency, while lower scores were observed for Competence (α = 0.69) and Character (α = 0.65). In the Spanish version, the five-factor structure also presented good overall fit based on CFA, χ^2^ (2, *N* = 768) = 11.75, *p* < 0.001, CFI = 0.987, RMSEA = 0.08.

The Multidimensional Anxiety Scale for Children (MASC; Salvador et al., 2017), which was used in the Portuguese sample, is a self-report questionnaire assessing the dimensions of anxiety in children and adolescents aged 8–19 years. It consists of 39 items measuring four dimensions: Physical symptoms, Harm avoidance, Social anxiety, and Separation anxiety. Participants rated each of the items using a 4-point scale anchored with the response options: 0 (never true about me), 1 (rarely true about me), 2 (sometimes true about me), and 3 (often true about me). The MASC translation has been validated for the Portuguese population (presenting good reliability; Tomé et al., 2019a).

The LAOM (Lestvica anksioznosti za otroke in mladostnike) anxiety scale (Kozina, 2012), used in the Slovenian sample, measures anxiety and three anxiety dimensions with 14 self-report items: Emotions—eight items (e.g., “I suddenly feel scared and I don’t know why”); Worries—three items (e.g., “I am very worried about my marks”); and Decision—three items (e.g., “I have difficulties making decisions”). It is designed to be used in school settings. Response categories are 1 (never), 2 (rarely), 3 (sometimes), 4 (often), and 5 (always). The dimension scores can be summed up to generate an overall anxiety score. The three-factor hierarchical structure was confirmed with CFA using the current sample [RMSEA = 0.079, CFI = 0.931, TLI = 0.908; Standardized Root Mean Square Residual (SRMR) = 0.045]. The internal consistency of the scale was adequate for Emotions (α = 0.867), Decision (α = 0.720) and Worries (α = 0.731) and for overall scale (α = 0.900).

The Generalized Anxiety Disorder-7 (GAD-7; Spitzer et al., 2006), used with Spanish participants, is a measure of generalized anxiety disorder. It is introduced by the question “How often have you been bothered by the following over the past 2 weeks?” and consists of seven items, which assess different anxious symptoms, following a 4-point-scale from 0 (not at all) to 3 (nearly every day). The overall score in this scale is calculated by adding the scores in each indicator, ranging from 0 to 21. Cut-off points have been established at 5, 10, and 15 for mild, moderate, and severe anxiety, respectively. The Spanish version has good reliability (α = 0.90), as shown by Gomez-Baya et al. (2019).

### Procedure

In Portugal, data were collected in 2018 in the context of a teacher training within the scope of a European funded project, EsCOOL (Tomé et al., 2019a). The paper-pencil format was used. The study was approved by the Lisbon University ethics committee (CHLN) as part of a larger postdoctoral research project. After obtaining parental permission for underage students, the data collection in Slovenia took place in 2017. Data were collected using a paper-pencil form. The questionnaires were administered by school psychologists. The time allocated to complete the questionnaire was 45 min (the majority of students finished earlier). The research design and data collection followed ethical guidelines of Slovenian Psychological Association. In Spain, the paper-pencil format was also used to collect data in 2017. The students individually and anonymously completed the self-report questionnaire, which took about 30 min, during normal class time. All students in selected school classes agreed to participate in the study and the university ethics committee (University of Huelva) gave approval.

### Data Analysis

First, we conducted correlational analyses to assess the bivariate associations between the 5Cs and anxiety. Then, we continued with multiple regression analyses with the enter method (entry variables were either the PYD global score or the individual 5Cs variables: Competence, Caring, Character, Confidence, and Connection). The target variable (anxiety and anxiety dimensions) was different across samples due to the measurement scales. Gender and age were added in regression models to control for their effects. IBM SPSS 25.0 software was used for the analyses.

## Results

We present the results for each country separately, starting with correlation coefficients between variables and then continuing with results from multiple regression analyses.

### Portugal

Table 1 shows the correlation coefficients between the global PYD variable, the 5Cs, and anxiety dimensions. The global PYD variable is significantly and positively correlated with Separation anxiety and Harm avoidance. The scales Harm avoidance and Separation anxiety are positively and (mostly) significantly associated with the 5Cs. For example, the highest positive coefficients are between Harm avoidance and the 5Cs (the highest between Confidence and Harm avoidance) and also between the 5Cs and Separation anxiety (the highest between Confidence and Separation anxiety). The correlations between the 5Cs and Social anxiety and between the 5Cs and Physical symptoms are mostly negative. The highest negative correlation coefficients can be found between Connection and Social anxiety, followed by Caring and Social anxiety, and then Competence and Physical symptoms.

**TABLE 1:** Correlations between the 5Cs and anxiety dimensions in the Portuguese sample.

	**Competence**	**Confidence**	**Character**	**Caring**	**Connection**	**PYD**
Competence	−					
Confidence	0.607**	−				
Character	0.439**	0.725**	−			
Caring	0.625**	0.606**	0.522**	−		
Connection	0.680**	0.562**	0.384**	0.712**	−	
PYD	0.809**	0.846**	0.745**	0.846**	0.839**	−
Anxiety	0.018	0.129**	0.167**	–0.008	0.017	0.078
Social	−0.110*	0.028	0.107*	−0.142**	−0.185**	–0.080
Physical	−0.138*	–0.043	0.010	–0.094	−0.110*	–0.092
Separation	0.156***	0.187**	0.172**	0.082	0.190**	0.196**
Harm	0.295**	0.376**	0.355**	0.243**	0.312**	0.388**

The 5Cs are competence, confidence, character, caring, and connection. The anxiety dimensions are Social anxiety, Physical symptoms, Separation anxiety, and Harm avoidance. PYD, positive youth development. *p < 0.05; **p < 0.001.

In multiple regression analysis, Caring is a significant and negative predictor of Social anxiety and Separation anxiety. Connection is a positive predictor of Separation anxiety and Harm avoidance, but a negative predictor of Social anxiety. Character is a significant and positive predictor of Social anxiety and Harm avoidance. Confidence is a significant and positive predictor of Harm avoidance, while Competence does not predict any of the anxiety dimensions. Age is a significant predictor of Physical symptoms and Harm avoidance (higher levels are reported in younger participants). Gender is a significant negative predictor of Physical symptoms (where females report higher levels of anxiety). With the included predictors, we can explain about 6.8% of the variance in Social anxiety, 5.6% in Physical symptoms, 5.7% in Separation anxiety, and about 18.6% in Harm avoidance (Table 2).

**TABLE 2:** The predictive power of the 5Cs for the MASC components of anxiety in the Portuguese sample.

	***B* (*SE*)**	***ß***	***t***	***R*^2^**	*R*^2^*
**Social anxiety, *F*(7, 376) = 4.994, *p* = 0.000**					
Constant	14.199 (3.810)				
Competence	−0.074(0.128)	–0.040	–0.543		
Confidence	0.170 (0.135)	0.103	1.232		
Character	0.270 (0.107)	0.201	2.742*		
Caring	−0.239(0.125)	–0.154	−1.977*		
Connection	−0.250(0.709)	–0.183	−2.332*		
Gender	−0.250(0.709	–0.019	–0.353		
Age	−0.041(0.148)	–0.014	–0.277	0.085	0.068
**Physical symptoms, *F*(7, 376) = 4.264, *p* = 0.000**					
Constant	8.337 (4.459)		1.879		
Competence	−0.249(0.150)	–0.124	–1.664		
Confidence	0.103 (0.159)	0.055	0.648		
Character	0.071 (0.125)	0.044	0.571		
Caring	−0.027(0.146)	–0.015	–0.188		
Connection	0.016 (0.106)	–0.012	0.148		
Gender	−2.350(0.829)	–0.155	−2.834*		
Age	0.639 (0.173)	0.191	3.697**	0.074	0.056
**Separation anxiety, *F*(7, 376) = 4.308, *p* = 0.000**					
Constant	3.533 (3.027)		1.167		
Competence	0.033 (0.102)	0.024	0.321		
Confidence	0.117 (0.108)	0.092	1.091		
Character	0.100 (0.085)	0.090	1.177		
Caring	−0.247(0.099)	–0.196	−2.485*		
Connection	0.223 (0.072)	0.249	3.100*		
Gender	−0.897(0.563)	–0.087	–1.593		
Age	−0.044(0.117)	–0.019	–0.371	0.074	0.057
**Harm avoidance, *F*(7, 376) = 13.514, *p* = 0.000**					
Constant	7.324 (2.409)		3.040*		
Competence	0.039 (0.081)	0.034	0.448		
Confidence	0.194 (0.086)	0.178	2.267*		
Character	0.158 (0.068)	0.166	2.328*		
Caring	−0.137(0.079)	–0.127	–1.734		
Connection	−0.175(0.057)	0.228	3.057*		
Gender	−0.882(0.448)	–0.100	–1.969		
Age	−0.189(0.093)	–0.097	−2.020*	0.201	0.186

The 5Cs are competence, confidence, character, caring, and connection. MASC, Multidimensional Anxiety Scale for Children. *p < 0.05; **p < 0.001.

### Slovenia

Table 3 shows the correlation coefficients between the global PYD variable, the 5Cs, and anxiety and its components (Emotions, Worries, and Decision). The global PYD variable is significantly and negatively associated with anxiety and its components Emotions and Decision. On the level of the distinct components of the 5Cs, the highest correlation is between anxiety (and its components) and Confidence, followed by correlations between anxiety (and its components) and Competence, and then between anxiety (and its components) and Connection, while the correlations between Character and anxiety (and its components) are lower and mostly non-significant. All of the correlations between the 5Cs and anxiety (and its components) are negative, except for Caring, for which all coefficients are significant and positive.

**TABLE 3:** Correlations between the global PYD factor, the 5Cs, and anxiety, and its components in the Slovenian sample.

	**Competence**	**Confidence**	**Character**	**Caring**	**Connection**	**PYD**
Competence	−					
Confidence	0.671*	−				
Character	0.347*	0.461*	−			
Caring	0.077	0.048	0.501*	−		
Connection	0.501*	0.579*	0.446*	0.191*	−	
PYD	0.707**	0.760**	0.768**	0.533**	0.785**	−
Anxiety	−0.379*	−0.476*	–0.081	0.245*	−0.326*	−0.271**
Emotions	−0.393*	−0.493*	–0.087	0.239*	−0.376*	−0.297**
Worries	−0.191*	−0.255*	–0.001	0.192*	−0.072*	–0.083
Decision	−0.321*	−0.386*	−0.105*	0.163*	−0.268*	−0.241**

The 5Cs are competence, confidence, character, caring, and connection. Emotions, Worries, and Decision are components of anxiety. PYD, positive youth development. *p < 0.05; **p < 0.001.

Table 4 presents the results from multiple linear regression for overall anxiety scores and the specific components of anxiety, with all 5Cs included as predictors. Confidence is a negative predictor of anxiety and all components of anxiety, while Connection is a negative predictor of anxiety and its components Emotions and Decision. Caring is a positive predictor of anxiety and all three components of anxiety. Age is a significant predictor of anxiety and its components Emotions and Worries (where older participants report lower levels of anxiety). With the included predictors, we can explain about 32.4% of the variance in anxiety and its component Emotions (35.1%), but a bit less variance in Decision (18.2%) and Worries (11%).

**TABLE 4:** The predictive power of the global PYD factor and 5Cs for anxiety and its components in Slovenian sample.

	***B* (*SE*)**	***ß***	***t***	***R*^2^**	*R*^2^*
**Anxiety, *F*(7, 391) = 28.200, *p* = 0.000**					
Constant	63.886 (5.617)		11.373**		
Competence	−0.317(0.156)	–0.116	–2.037		
Confidence	−0.802(0.171)	–0.308	−4.684**		
Character	0.160 (0.140)	0.064	1.139		
Caring	0.659 (0.118)	0.283	5.610**		
Connection	−0.368(0.282)	–0.175	−3.241*		
Gender	−0.055(0.089)	–0.026	–0.616		
Age	−0.716(0.282)	–0.107	−2.544*	0.335	0.324
**Emotions, *F*(7, 393) = 31.902, *p* = 0.000**					
Constant	38.573 (3.505)		11.006**		
Competence	−0.180(0.097)	–0.104	–1.855		
Confidence	−0.516(0.106)	–0.312	−4.854**		
Character	0.124 (0.087)	0.078	1.423		
Caring	0.412 (0.073)	0.277	5.633**		
Connection	−0.301(0.071)	–0.224	−4.253**		
Gender	−0.028(0.055)	–0.020	–0.501		
Age	−0.492(0.176)	–0.116	−2.802*	0.362	0.351
**Worries, *F*(7, 394) = 8.079, *p* = 0.000**					
Constant	12.535 (1.629)		7.695		
Competence	−0.063(0.045)	–0.091	–1.395		
Confidence	−0.127(0.050)	–0.192	−2.549*		
Character	0.017 (0.041)	0.027	0.427		
Caring	0.128 (0.034)	0.215	3.737*		
Connection	0.007 (0.033)	0.012	0.197		
Gender	−0.012(0.026)	–0.022	–0.469		
Age	−0.191(0.082)	–0.114	−2.334*	0.126	0.110
**Decision, *F*(7, 396) = 13.777, *p* = 0.000**					
Constant	13.292 (1.565)		8.496**		
Competence	−0.074(0.043)	–0.107	–1.711		
Confidence	−0.153(0.048)	–0.231	−3.215*		
Character	0.015 (0.039)	0.023	0.377		
Caring	0.122 (0.033)	0.205	3.730**		
Connection	−0.075(0.032)	–0.140	−2.387*		
Gender	−0.015(0.025)	–0.027	–0.584		
Age	−0.062(0.078)	–0.036	–0.788	0.196	0.182

The 5Cs are competence, confidence, character, caring, and connection. emotions, worries, and decision are components of anxiety. PYD, positive youth development. *p < 0.05; **p < 0.001.

### Spain

The correlation coefficient between the global PYD variable and anxiety is both significant and negative (Table 5). Regarding the 5Cs, the correlation coefficients between the 5Cs and anxiety are all significant and negative, except for Caring, which shows a positive correlation.

**TABLE 5:** Correlation coefficients between the 5Cs and anxiety in the Spanish sample.

	**Competence**	**Confidence**	**Character**	**Caring**	**Connection**	**PYD**
Competence	−					
Confidence	0.517**	−				
Character	0.158**	0.369**	−			
Caring	0.019	0.083**	0.542**	−		
Connection	0.328**	0.480**	0.436**	0.273**	−	
Anxiety	−0.133**	−0.298**	−0.076**	0.097*	−0.201**	−0.172**

The 5Cs are competence, confidence, character, caring, and connection. PYD, positive youth development. *p < 0.05; **p < 0.001.

Based on multiple regression analysis, Competence and Caring are both significant and positive predictors of anxiety, while Confidence and Connection are both significant and negative predictors of anxiety (Table 6). Gender is a significant and positive predictor of anxiety (where females relative to males report higher levels of anxiety). With the included predictors, we can explain about 13.8% of the variance in anxiety.

**TABLE 6:** The predictive power of the 5Cs for anxiety in the Spanish sample.

	***B* (*SE*)**	***ß***	***t***	***R*^2^**	*R*^2^*
**Anxiety, *F*(7, 660) = 16.207, *p* > 0.001**					
Constant	14.305 (2.408)		5.939**		
Competence	0.894 (0.342)	0.112	2.614*		
Confidence	−2.044(0.384)	–0.250	−5.324**		
Character	−0.407(0.531)	–0.036	–0.766		
Caring	0.929 (0.321)	0.126	2.899*		
Connection	−1.464(0.417)	–0.157	−3.513**		
Gender	2.007 (0.417)	0.188	4.814**		
Age	−0.127(0.083)	–0.055	–1.529	0.147	0.138

The 5Cs are competence, confidence, character, caring, and connection. *p < 0.05; **p < 0.001.

## Discussion

In the present study, we examined the association between the global PYD factor (and the 5Cs) and anxiety (and its dimensions). We analyzed the associations in three different contexts (Portugal, Slovenia, and Spain) using three different anxiety measures as outcomes and the same PYD measure as the predictor of these outcomes. Given that the anxiety measures are different, direct comparisons across contexts are not possible. Nevertheless, they are at the same time informative for individual contexts as well as for the future research directions. The underlying goal was to inform the contextualized prevention and intervention measures needed in all three contexts. We separately investigated the associations between anxiety and PYD as a global PYD factor and as distinct components of the 5Cs.

In Slovenia and Spain, the association between the global PYD factor and the total anxiety score was significant and negative, in line with the PYD theoretical assumption where the 5Cs are associated with a positive contribution and fewer behavioral and emotional difficulties (Lerner, 2007). Similar findings for the global PYD score were obtained in research in the United States (Geldhof et al., 2014) and Europe (Erentaitë and Raižienë, 2015; Holsen et al., 2017). In Portugal, the association between the PYD global score and the anxiety total score was negative, the same as in Slovenia and Spain, but not significant. This could be due to the different patterns of associations of different dimensions of anxiety with the global PYD score (two were associated positively and two negatively with the global PYD score). On the level of the 5Cs, we observed different patterns and some similarities across contexts. For instance, in Slovenia and Spain, confidence and connection were significantly and negatively predicting anxiety. This means that the more confident and connected youth were, the less anxiety they reported. The opposite was true for caring in Slovenia and Spain: the more caring youth were, the higher their reported level of anxiety.

The negative association between anxiety and caring is aligned with previous research (Geldhof et al., 2014; Truskauskaitë-Kunevičienë et al., 2014; Holsen et al., 2017) and can become clearer when looking into definitions of caring as combination of empathy and sympathy in the context of PYD (Geldhof et al., 2013). Empathy is a multidimensional process that combines physiological, emotional, and cognitive components together with a metacognitive awareness that distinguishes between one’s own emotional state and the emotional state of the other (Hoffman, 2008). What metacognitive awareness does, it helps individuals to distinguish between their own emotional arousal and the emotional arousals of others (Hoffman, 2008). It is therefore possible that people who are characterized as more caring would have difficulties with emotional contagion and are not able to distinguish between another person’s arousal and their own arousal. For example, when an individual observes another person’s emotion, he or she may feel emotional stress (an emotional component of empathy), and this emotional stress can lead to prosocial behavior to help the other person (making empathy very important here). However, the individual may override the arousal of the other person, a condition known as empathic over-arousal (Hoffman, 2008), when he or she shifts attention to his or her own emotional stress and does not help the other person who was originally under stress. Thus, the individual is mainly concerned with his or her own emotional arousal and tries to avoid the other person and the situation. This attentional shift can result in a higher anxiety level, as shown in the Slovenian and Spanish data. In Portugal, caring is a negative predictor of anxiety as it would be expected based on the PYD framework (Lerner, 2007). We assume that either the characteristics of caring differ (e.g., containing more metacognitive awareness) or the association depends on the dimension of anxiety measured in different contexts. More insight into the complexity of the relationship between caring and anxiety is provided by the dimensions of anxiety (available in the Slovenian and Portuguese data). In Slovenia, caring is a significant and positive predictor of all three components of anxiety (emotions, worries, and decision). In Portugal, caring is a significant and negative predictor of social anxiety and separation anxiety. The more caring youth in Portugal are, the lower their social and separation anxiety. These two types of anxiety are the ones strongly linked to interpersonal relationship where caring, as a combination of empathy and sympathy, plays a crucial role, e.g., more empathy and sympathy is associated with more positive relationships with others (Eisenberg et al., 2010). More positive relationships with others provide more security in attachment (Arslan et al., 2012) and therefore lower separation anxiety.

As for the other Cs, the more confident youth in Portugal were, the higher the level of their anxiety (harm avoidance) and the more of high character youth in Portugal were the higher was their anxiety (social anxiety and harm avoidance). In case of confidence this is the opposite of what was observed in Slovenia and Spain. In our preliminary hypothesis of character, we focused on its definition, e.g., possession of standards for correct behavior in relation to social and cultural norms. High character can in this sense reflect high anxiety associated with breaking the rules. One of the characteristics of anxious individuals is fear of evaluation, this is especially true for social anxiety (Kocovski and Endler, 2000). Social anxiety was also included only in the Portuguese sample and the findings can reflect this association. As for confidence, the explanations is not as straight forward since we expected high levels of confidence to be associated with low levels of anxiety (van Tuijl et al., 2016), as it was in Slovenia and Spain. The relationship between confidence (self-efficacy and self-worth) and anxiety has been widely researched indicating that lower confidence and especially emotional self-efficacy (Mathews et al., 2016) is related to higher levels of anxiety (Voight et al., 2000; Soleimani et al., 2017). In case of Portugal, we would stress here the need for future research to look more into details of this relationship.

Despite the differences in the pattern of relationships between PYD and anxiety, e.g., caring and anxiety, confidence and anxiety, across countries there is also a common ground. Specifically, connection is significantly and inversely related to anxiety in Slovenia, Spain, and Portugal (higher connection predict lower social anxiety). The more connected youth are the lower their anxiety. In Slovenia, on the anxiety dimension level connection is a negative predictor of its components, emotion and decision. In Portugal, we gained more insight into the relationship between connection and anxiety with inclusion of the clinical measure; this approach revealed different associations between the 5Cs and the dimensions of anxiety. For example, connection is a negative predictor of social anxiety while it is a significant positive predictor of separation anxiety and harm avoidance, the latter indicating the complexity of connection. Thus, similar to caring, the characteristics of connection are probably important and need to be evaluated in future studies. One concept that could be added to future research is the attachment style: even though unsecure attachment is reflected in strong connection; it could give rise to separation anxiety. In cases where the individuals are safely attached and connected, their social anxiety is lower. Because the measure used in the Portuguese sample is clinical, the findings present a starting point for further analyses of the role PYD can play in a clinical setting. Even though the relationship between social connectedness and anxiety is inverse and significant across all developmental stages, it is more prominent in adolescence (Levula et al., 2018). Therefore, in intervention programs it is important to include as many resources in the ecology of youth (such as families, school, neighborhood, and the local community) that can support adolescents’ development in more positive directions and in this way lower their anxiety as it is the case in whole school approach used in Portugal (Tomé et al., 2021) and in Slovenia (e.g., the HAND in HAND, Kozina, 2020).

## Practical Implications and Conclusion

Based on the PYD perspective (Lerner et al., 2011), positively developing youth should have lower levels of problem behaviors and also demonstrate an enhanced potential to contribute actively to personal and societal well-being, which is also reflected in lower anxiety. This is to some extent (if we ignore caring) true for the Slovenian and Spanish data. Overall, the findings are in line with researchers who argue that the associations between a global PYD factor and the 5Cs with behavioral and emotional difficulties are not simply inverse (Lerner et al., 2015). On the level of 5Cs, our findings reveal the complexity of adaptive regulations as highlighted by Geldhof et al. (2019), indicating that the associations between the 5Cs and anxiety depend on the context. Thus, we see that going from the level of the global PYD factor to the level of the distinct components of the 5Cs brings new insights, including an interesting, previously reported finding regarding caring (Geldhof et al., 2014; Truskauskaitë-Kunevičienë et al., 2014; Holsen et al., 2017) in Slovenia and Spain. Based on the findings in Slovenia and Spain we suggest addressing anxiety by using strategies that nurture confidence and connection but would remain cautious with the promotion of caring. In Spain, we would promote the ongoing programs that already support the psychological well-being (e.g., the Happy Classrooms Programme) but would advise boosting activities related to confidence and connection. In Slovenia, PYD interventions are still in planning phase; therefore, these findings can be incorporated and the effects and the role each of the 5Cs play in PYD in Slovenia can be evaluated in detail. Some of the existing programs targeting social and emotional competencies (more associated with caring, connection and competence) can be upscaled at the systemic level with addition of activities targeting character and confidence. In Portugal, promoting caring can be beneficial for the prevention of social and separation anxiety. There are several ongoing programs in Portugal that already include the empathy component and we would encourage their further use (for review, see Tomé et al., 2018, 2019a). Thus, we would argue that not only the quality of caring (e.g., the level of metacognitive awareness) and the dimension of anxiety, but also the context in which the relationship is investigated is important. Last but not least our findings confirm the importance of gender and age in the development of anxiety (Furr et al., 2009). Aligned with the literature (Furr et al., 2009) girls are more prone to higher anxiety in Spain and in Portugal and would need more attention in prevention and intervention programs in these two contexts. As for age it turned out to be significant positive predictor of anxiety in Portugal (positive of physical symptoms and negative predictor of harm avoidance and social anxiety) and in Slovenia. Since the age of the samples used in different contexts vary a lot the findings are not comparable although we stress the need to consider age as important variables in future studies as well.

### Limitations and Future Directions

Despite revealing the rich complexity of the relationship between PYD and anxiety, and the roles the contexts as well as different components of anxiety can play in that relationship, the current findings are still based on convenience samples and are cross-sectional, which limits our conclusions. We are also limited in making comparisons across samples because of the different anxiety measures (clinical and non-clinical) and the different age ranges of the participants. Nevertheless, these differences can also be seen as an added value indicating that PYD and anxiety are significantly associated using different anxiety measures measuring different anxiety dimensions in different age groups. Our findings, even though robust considering that we used different scales and made similar observations to some extent, are not suitable for comparison across countries. Thus, we recommend similar age groups and measurement across countries in future studies that would also enable multigroup analysis. Nevertheless, the current study can be used as a starting point and can to some extent guide efforts in the prevention of anxiety in specific contexts. For example, in Slovenia, there are currently no specific PYD-framed interventions; therefore, the findings can be of use as a building block. In addition, specific information is provided by the inclusion of different multidimensional anxiety measures that provide contextual data. In terms of research, our findings support the common ground for intervention in all three contexts, that is, promotion of connection but also special care when it comes to caring. We found a positive relationship between caring and anxiety and its dimensions in Slovenia and Spain, indicating that high levels of caring are not always optimal, and the association probably depends on the specific characteristics of caring. Similar ambiguity was detected in Portugal regarding connection, indicating that not only the amount of connection but also the quality of connection likely plays a role. In future research, we would therefore support the more detailed measurement of all 5Cs and related constructs to reveal the complete complexity of positive adaptive regulations, preferably using representative samples in a longitudinal design.

### Key Messages

Our findings aim to encourage professionals and public policies to take into account the importance of being aware of the development and promotion of the 5Cs in adolescence, namely in schools and in educational settings. Promoting connection appears to play important role that needs to be investigated further, and our findings, even though cross-sectional support the inclusion of connection in all school-based programs in the three studied countries. The study has the advantage of having gathered a large international research group in which additional qualitative research would be needed in order to understand what exactly the 5Cs (especially caring) mean in different contexts (countries, age groups), and what is considered an optimal level of caring for each sociocultural context or situation (e.g., with focus groups or semantic differentiators). A key message for professionals and for public politics is that PYD enhanced by social and emotional competencies may be central to the health and well-being of youth across Europe. However, recommendations must keep in mind that cultural expressions of these competencies vary and have to be included in prevention and intervention strategies.

## Data Availability Statement

The raw data supporting the conclusions of this article will be made available by the authors, without undue reservation.

## Ethics Statement

The studies involving human participants were reviewed and approved by the Lisbon University Ethics Committee, the University of Huelva Ethics Committee and followed ethical guidelines of Slovene Psychological Association. Written informed consent to participate in this study was provided by the participants’ legal guardian/next of kin.

## Author Contributions

AK conducted the analysis across the three contexts, drafted the manuscript, designed the study, and collected the data in Slovenia. DG-B designed the study and collected the data in Spain. GT and MG designed the study and collected the data in Portugal. NW initiated the research on PYD across the countries. All authors were involved in the critical review and the revision of the manuscript, and approved the final version of the manuscript.

## Conflict of Interest

The handling editor was organizing a Research Topic with one of the authors [NW]. The remaining authors declare that the research was conducted in the absence of any commercial or financial relationships that could be construed as a potential conflict of interest.
